# Large-Area Perovskite Solar Module Produced by Introducing Self-Assembled L-Histidine Monolayer at TiO_2_ and Perovskite Interface

**DOI:** 10.3390/nano14151315

**Published:** 2024-08-04

**Authors:** Hung-Chieh Hsu, Jung-Che Tsao, Cheng-Hsien Yeh, Hsuan-Ta Wu, Chien-Te Wu, Shih-Hsiung Wu, Chuan-Feng Shih

**Affiliations:** 1 Department of Electrical Engineering, National Cheng Kung University, Tainan 70101, Taiwan; n28084020@gs.ncku.edu.tw (H.-C.H.); jerry881015@gmail.com (J.-C.T.); n28084012@mail.ncku.edu.tw (C.-H.Y.); 2 Green Energy and Environment Research Laboratories, Industrial Technology Research Institute, Tainan 711010, Taiwan; 3 Applied High Entropy Technology (AHET) Center, National Cheng Kung University, Tainan 70101, Taiwan; 4 Department and Institute of Electrical Engineering, Minghsin University of Science and Technology, Hsinchu 30401, Taiwan; htwu@must.edu.tw; 5 Symbio, Inc., New Taipei City 241457, Taiwan; ctwu168@gmail.com; 6 Hierarchical Green-Energy Materials (Hi-GEM) Research Center, National Cheng Kung University, Tainan 70101, Taiwan

**Keywords:** perovskite, self-assembled monolayer, large area, L-histidine

## Abstract

Perovskite solar cells have been proven to enhance cell characteristics by introducing passivation materials that suppress defect formation. Defect states between the electron transport layer and the absorption layer reduce electron extraction and carrier transport capabilities, leading to a significant decline in device performance and stability, as well as an increased probability of non-radiative recombination. This study proposes the use of an amino acid (L-Histidine) self-assembled monolayer material between the transport layer and the perovskite absorption layer. Surface analysis revealed that the introduction of L-Histidine improved both the uniformity and roughness of the perovskite film surface. X-ray photoelectron spectroscopic analysis showed a reduction in oxygen vacancies in the lattice and an increase in Ti^4+^, indicating that L-Histidine successfully passivated trap states at the perovskite and TiO_2_ electron transport layer interface. In terms of device performance, the introduction of L-Histidine significantly improved the fill factor (FF) because the reduction in interface defects could suppress charge accumulation and reduce device hysteresis. The FF of large-area solar modules (25 cm^2^) with L-Histidine increased from 55% to 73%, and the power conversion efficiency (PCE) reached 16.5%. After 500 h of aging tests, the PCE still maintained 91% of its original efficiency. This study demonstrates the significant impact of L-Histidine on transport properties and showcases its potential for application in the development of large-area perovskite module processes.

## 1. Introduction

Perovskite solar cells, characterized by a low carbon footprint and continuously improving conversion efficiencies, have emerged as one of the most important photovoltaic devices [1]. The current highest efficiency of perovskite solar cells reaches 26.7% [2]. However, organic perovskite materials are prone to rapid degradation due to humidity and temperature fluctuations, and the formation of interface defects between the perovskite and transport layers remains a major bottleneck in improving the efficiency and lifespan of perovskite devices [3,4]. Therefore, many research teams have recently begun investigating the use of self-assembled monolayers (SAMs) as passivation materials to suppress interface defects, enhance interactions between interfaces [5,6], improve energy level matching between the absorption and transport layers, and reduce the probability of carrier recombination at the interface, thereby enhancing the charge extraction capabilities of the device. D. S. Mann et al. introduced 3-(Triethoxysilyl)propylamine (TSPA) to the transport layer interface of inverted (p-i-n) perovskite solar cells. TSPA passivates hydroxyl groups on the NiOx surface, thereby improving interface defects [7]. F. Han et al. employed bifunctional 4-picolinic acid (4-PA) to modify the electron transport layer/perovskite interface. The COOH group of 4-PA forms a chemical bond with mp-TiO_2_, which enhances electron transport, reduces charge accumulation at the interface, and mitigates device hysteresis, resulting in an increase in PCE from 14.65% to 18.9% [8]. Y. C. Shih et al. introduced an amino acid material, glycine, to modify the TiO_2_/CH_3_NH_3_PbI_3_ heterointerface, which improves defect states generated during the perovskite crystallization process, leading to an increase in PCE from 8.35% to 12.02% [9]. These materials mainly inhibit defects in either the perovskite or electron transport layer. A summary of SAM materials is presented in Table S1. However, L-His can form hydrogen bonds with perovskite organic cations, and its carboxyl groups can anchor to the mesoporous TiO_2_ surface, passivating defects in both the electron transport layer and the perovskite simultaneously. Additionally, SAMs have been reported to influence perovskite crystallinity, favoring the nucleation and growth of perovskite films and promoting the formation of dense perovskite layers [10,11]. This contributes to the improved optoelectronic properties, stability, and lifespan of the devices [12,13,14,15]. H. B. Kim et al. found that introducing carboxylic acid molecules on the surface of the mesoporous TiO_2_ layer enhances its wettability and the adhesion capability of the carboxylic acid molecules to the TiO_2_ layer [16]. In the perovskite structure, carboxylic acid molecules can stably anchor to the surface of the mesoporous TiO_2_ layer, reducing trap states at the transport layer interface and increasing charge extraction efficiency [17,18].

This study introduces L-Histidine (L-His) as a surface passivation material. L-His is composed of amino (-NH_2_) and carboxyl (-COOH) functional groups, forming a self-assembled material that can improve the interfacial properties between the electron transport layer and the perovskite. X. Sun et al. introduced L-His as a self-assembled material for the SnO_2_/perovskite interface. L-His enhanced the interaction between the transport layer and the perovskite interface and adjusted the energy-level structure [19]. In this study, L-His was incorporated into a meso-type perovskite device. The soaking method was used to deposit the L-His self-assembled layer, making this approach suitable for developing large-area modules. It was found that the -NH_2_ group can form hydrogen bonds with MA ions, while the carboxyl group can fill oxygen vacancies in the TiO_2_ electron transport layer. The hydrogen atom in the imidazole ring provides lone-pair electrons, which passivate uncoordinated Pb^2+^ defects in the perovskite film [20], thereby enhancing the stability and aging properties of the perovskite film. In addition, the perovskite solar cells were fabricated using a blade-coating process, and single perovskite cells were integrated using picosecond laser technology (P1, P2, P3). The results showed significant improvements in the open-circuit voltage (Voc), fill factor (FF), and power conversion efficiency (PCE). Analyses using the dark current curve, photoluminescence (PL), X-ray photoelectron spectroscopy (XPS), and nuclear magnetic resonance (NMR) confirmed that the interfacial properties between the electron transport layer and the perovskite layer were improved. Finally, a large-area solar module (25 cm^2^) was successfully fabricated, with the FF increasing from 55% to 73% and a PCE of 16.5%. After 500 h of aging tests, the PCE still maintained 91% of its original efficiency.

## 2. Materials and Methods

### 2.1. Materials

Methylammonium iodide (MAI) was purchased from GreatCell Solar. Lead (II) iodide (PbI_2_, 99.9985%) was purchased from TCI. Spiro-OMeTAD (>99.5%) was purchased from Derthon. Dimethyl sulfoxide (DMSO, 99%) and dimethylformamide (DMF) were purchased from J.T. Baker. All chemicals were used as received. The perovskite precursors were prepared in a N_2_-filled glove box, where oxygen was < 1 ppm and relative humidity was <30%. The perovskite precursor solution was composed of DMF and DMSO at a ratio of 8:2, and the solvent was mixed and stirred evenly. The solution was then filtered through a 0.25 µm filter sieve.

### 2.2. Device Fabrication

The laser process for producing large-area modules is divided into three stages (P1, P2, P3). The power density calculated by the power meter was fixed at 5.25 W, and the laser scanning speed was set at 2000 mm/s. First, the P1 process to scribe FTO was performed on the substrate. A TiCl_4_ solution was used to fabricate a compact TiO_2_ layer on the FTO. The mesoporous TiO_2_ layer was prepared from a diluted TiO_2_ slurry using a doctor blade coater on the dense TiO_2_ layer of the substrate. The TiO_2_-coated substrates were annealed in a high-temperature furnace at 500 °C for 60 min. Self-assembled monolayers prepared by the soaking method are more suitable for the large areas application than those fabricated by the spin-coating method. The perovskite precursor solution, MAPbI_3_, was prepared and coated on the surface of the substrate. Subsequently, a Spiro-OMeTAD solution was spin-coated onto the perovskite film within a dry-air glove box with a relative humidity of <10%. Following the deposition of various layers, the P2 process was executed to remove the films stacked on the FTO. Subsequently, an electrode layer (Au) was deposited, and the P3 process was performed to ablate all layers except for FTO, thereby completing the perovskite module.

### 2.3. Characterizations

The microstructures of the perovskite thin films were analyzed by scanning electron microscope (SEM, Hitachi SU8000, Tokyo, Japan). The nanoscale imaging of surface profiles was carried out by an atomic force microscope (AFM) (Bruker Dimension Icon). The crystalline structures were characterized by X-ray diffraction (XRD, Bruker D8 Discover, Karlsruhe, Germany) with an angular resolution of 0.02° and Cu-Kα radiation. The transmittance spectra of thin films were collected by using a HITACHI U-2800 UV–vis–NIR spectrophotometer (Tokyo, Japan). The space-charge limit current (SCLC) was analyzed by current-voltage (I–V) measurements using Keithley 2400 in the dark. Current density–voltage (J–V) curves were generated by using a solar simulator (Enlitech SS-F5-3A) at AM 1.5G and 100 mW/cm2 illumination and a source meter (Keithley 2400) with a scan range from +1.1 V to −0.1 V and a scan rate of 0.05 V/s. The steady-state photoluminescence (PL) spectra and time-resolved photoluminescence (TR-PL) spectra of perovskite films were measured by using a Princeton Instruments Acton 2150 spectrophotometer. X-ray photoelectron spectroscopy (XPS) (PHI VersaProbe 4) measurements were performed with Al Kα emission. ^1^H NMR spectra were measured on Bruker AVANCE III HD 600MHz NMR Spectrometer.

## 3. Results and Discussion

**Figure 1:**
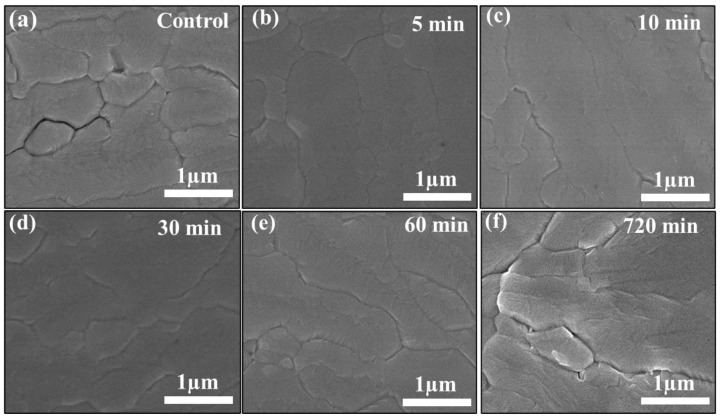
SEM images of perovskite films deposited on L-His with different soaking times: (**a**) 0 min (control), (**b**) 5 min, (**c**) 10 min, (**d**) 30 min, (**e**) 60 min, and (**f**) 720 min.

**Figure 2:**
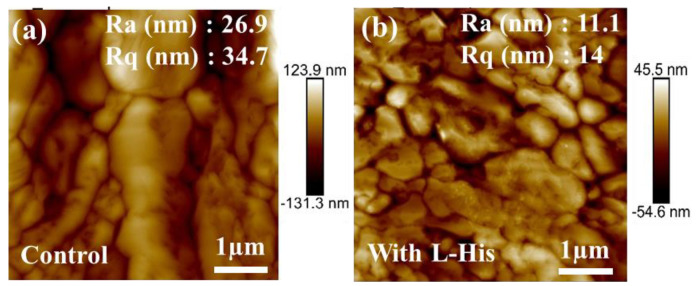
Atomic force microscopy images: (**a**) control; (**b**) with L-His.

L-His films were prepared using a soaking method. Figure 1a shows the control group, while Figure 1b–f depict the morphologies of perovskite films deposited on L-His for varying soaking times. After soaking for 5 min, the perovskite surface on L-His exhibited significantly reduced pore formation at grain boundaries compared to the control, with noticeably larger grain sizes and fewer pores. After 12 h of soaking time, the surface morphology became less dense, with pore formation at the edges of the grains. Figure 2a,b present the surface roughness analysis, indicating that the root-mean-square roughness of the perovskite film surface decreased from 34.7 nm to 14 nm upon introducing L-His. SEM images of perovskite films deposited on L-His with different concentrations are shown in Figure S1. The charge-transfer capability and adhesion between the perovskite layer and the hole transport layer (spiro) are influenced by the surface roughness of the perovskite film. Therefore, a low surface roughness improves the compatibility of the absorber layer and the hole transport layer interface, enhancing device performance. Contact angle analysis revealed a decrease in contact angle from 22.9° in the original control group to 21.1° after introducing L-His at the interface of the transport layer, as shown in Figure S2. This indicates that the improved surface wettability from L-His significantly contributes to the growth of perovskite grains. A decrease in the contact angle indicates that the TiO_2_ surface exhibits better hydrophilicity, facilitating the more uniform deposition of the perovskite precursor solution on the TiO_2_ surface. This improves the quality of the perovskite film, promoting increased grain size and a more densely packed morphology.

**Figure 3:**
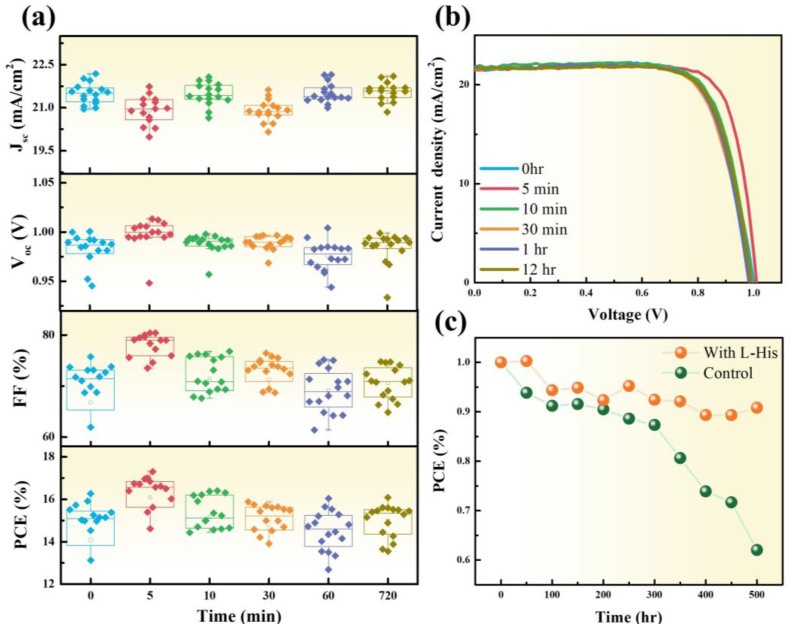
(**a**) The I-V characteristics of the perovskite solar cell after different soaking times. (**b**) The I-V curve of the perovskite solar cell. (**c**) The aging test of perovskite solar cells with and without (control) L-His.

Figure 3a,b analyze the influence of different soaking times of L-His on device performance. The optimized concentration of the L-His solution is 0.6 mM, with soaking times of 0, 5, 10, 30, 60, and 720 min. The photovoltaic performance of perovskite solar cells with different L-His deposition times is shown in Figure S3. The trend of device characteristics with varying concentrations of L-His is shown in Figure S4a,b. Figure S4c shows that the introduction of L-His will not affect the transmittance properties of perovskite devices. Observing the box plots, it is noted that after soaking for 5 min, both the FF and PCE significantly increase. The increase in the FF suggests that the introduction of L-His improves the interface quality between the transport layer and the absorber layer [21,22]. However, the short-circuit current (Jsc) decreases. This decrease in Jsc is attributed to the deposition of L-His on the transport layer, forming a thin passivation layer that slightly inhibits current characteristics. Figure 3c illustrates the aging trend of the devices in an atmospheric environment after the addition of L-His. M. Hou et al. used dopamine (DA) to modify the SnO_2_ transport layer/perovskite interface, resulting in perovskite photovoltaic devices that retained 80% of their initial efficiency after 300 h [23]. C.-T. Lin et al. introduced amino acid derivatives into MAPbI_3_ perovskite solar cells, reducing oxygen-induced photodegradation and maintaining 78% of their initial efficiency after 120 h in an atmospheric environment [24]. Y. C. Shih et al. used glycine to modify the TiO_2_/CH_3_NH_3_PbI_3_ heterointerface, improving crystallization defects and retaining 78% of their initial efficiency after 840 h in an atmospheric environment (RH ≤ 30%) [18]. In this study, after 500 h of aging testing, the PCE remained at 91% of its original efficiency.

**Figure 4:**
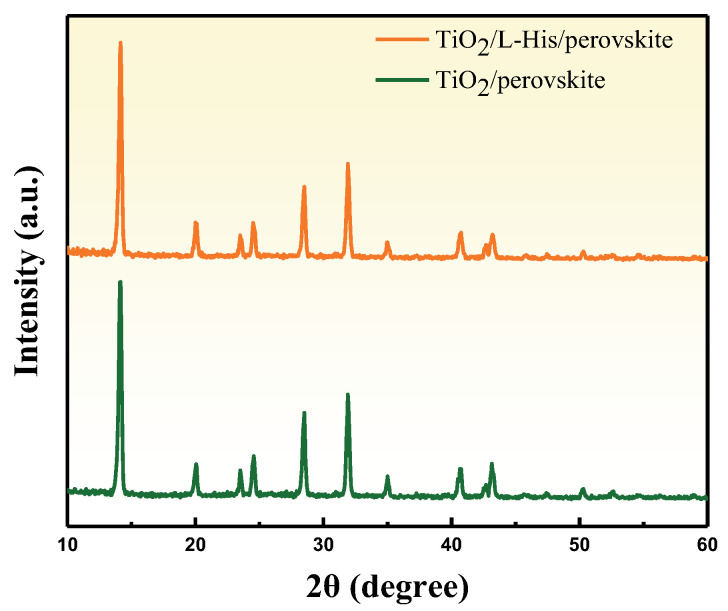
XRD patterns of perovskite film with and without L-His.

**Figure 5:**
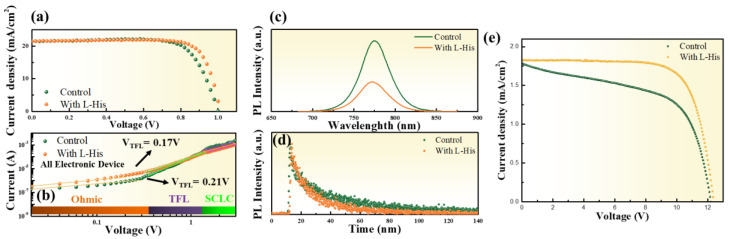
(**a**) I-V curve, (**b**) dark current curve, (**c**) PL, (**d**) TRPL spectra of perovskite solar cells with and without L-His, (**e**) I-V curve of perovskite solar module.

Figure 4 shows the XRD diffraction peak patterns, with the main characteristic peaks of the perovskite at (110), (220), and (310) and diffraction peak angles of 14.08°, 28.4°, and 31.9°, respectively. After adding L-His, no other secondary phases were observed in the diffraction patterns, and the intensity of the main characteristic peaks was similar to the original, indicating that the introduction of L-His does not affect the perovskite structure. This is crucial for the long-term stability of the perovskite absorber layer. Figure 5a displays the illuminated IV curves of the perovskite devices. After adding L-His, the FF increased from 75% to 79%, and the PCE rose from 16.26% to 17.3%. The characteristics of the perovskite devices are listed in Table 1.

Figure 5b shows the I-V characteristics in the dark to analyze the defect density and trap-filled limit voltage (V_TFL_) of devices with only the electron transport layer structure. Three regions can be observed in the figure: the ohmic region (I ∝ V), the trap-filled region (I ∝ V^n^, n > 2), and the SCLC region (I ∝ V^2^). In the trap-filled region, the current exhibits a rapid nonlinear increase, indicating that all trap states are filled with injected carriers; the voltage corresponding to the intersection of the ohmic and trap-filled regions is defined as the trap-filled limit voltage (V_TFL_). The defect density of the perovskite film can be calculated using the following equation: V_TFL_ = (e × N_t_ × d^2^)/(2 × ε × ε_0_), where e, N_t_, d, ε, and ε_0_ represent the electron charge, trap density, perovskite film thickness, perovskite dielectric constant, and dielectric constant of free space, respectively [25]. TiO_2_/L-His/MAPbI_3_ has a lower V_TFL_ = 0.17 V and N_t_ = 3.96 × 10^1^⁵ cm^−3^, indicating a lower defect density in the electron structure. This suggests that introducing L-His at the interface between the electron transport layer and the perovskite helps suppress defects. After introducing L-His, the V_TFL_ significantly decreased from 0.21 V to 0.17 V. The N_t_ values of TiO_2_/MAPbI_3_ and TiO_2_/L-His/MAPbI_3_ are 4.66 × 10^1^⁵ cm^−3^ and 3.96 × 10^1^⁵ cm^−3^, respectively. The V_TFL_ and N_t_ of the two structures are listed in Table S2.

In analyzing the reasons for the decrease in the FF and PCE with increasing L-His soaking time, it was found that with an L-His soaking time of up to 24 h, the V_TFL_ increased from 0.21V to 0.33V, and the trap density rose from 3.96 × 10^1^⁵ cm^−3^ to 7.36 × 10^1^⁵ cm^−3^, as shown in Figure S5. The results indicate that prolonged soaking in L-His increases the defect density in the device, thereby affecting its electrical properties. From Figure 5c, the PL spectra clearly show a significant decrease in PL peak intensity after introducing L-His. The substantial quenching of PL intensity indicates an improved electron extraction capability of the transport layer after introducing L-His. Radiative recombination between trap states at the perovskite/electron transport layer interface can cause band bending on the film surface and a red shift in the diffraction peak [26]. The slight blue shift of the PL peak from 775 nm to 771 nm in the perovskite film with L-His indicates an effect of passivating trap states at the perovskite/electron transport layer interface [27].

Next, we analyzed the impact of L-His on the carrier lifetime using time-resolved photoluminescence (TRPL). Solar cells with electron transport layer/perovskites made of the following structures were analyzed: (a) TiO_2_/MAPbI_3_ and (b) TiO_2_/L-His/MAPbI_3_. The TRPL spectra were fitted using a bi-exponential decay model to obtain the PL carrier lifetime: I(t) = A1 exp(−t/τ1) + A2 exp(−t/τ2) + I_0_. A1 and A2 are decay amplitudes, and τ is the decay time constant. The fast decay lifetime (τ1) represents non-radiative recombination caused by trapping processes when charges pass through the perovskite surface, while the slow decay lifetime (τ2) is associated with radiative recombination processes occurring in the bulk perovskite [28]. The quenching of the fast decay lifetime indicates the effective suppression of non-radiative recombination through the passivation of interface defects in the perovskite; the quenching of the slow decay lifetime indicates longer charge transport distances after charge separation. From the TRPL spectra in Figure 5d, it can be corroborated that the electron extraction capability of the transport layer is enhanced. Without L-His, a longer PL lifetime is shown, while the average carrier lifetime decreases from 56.4 ns to 24.4 ns after introducing L-His, indicating faster carrier extraction into the transport layer and reduced radiative recombination [29]. The significant quenching of the TRPL lifetime is attributed to the introduction of L-His, facilitating faster carrier transfer at the interface [30]. The TRPL results are summarized in Table S3.

**Figure 6:**
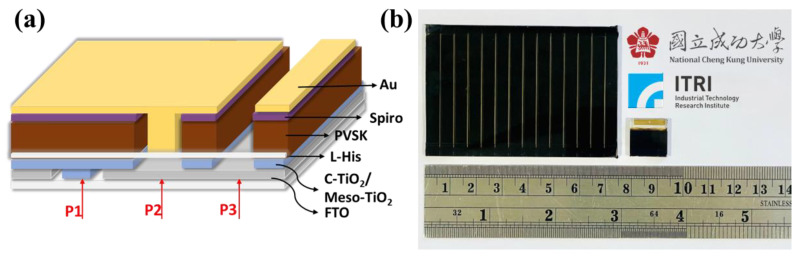
(**a**) Schematic of module; (**b**) photograph of perovskite solar module and single cell.

Figure 5e demonstrates the performance of large-area perovskite modules combined with L-His, with the perovskite solar module consisting of 12 single cells connected in series. The figure shows that the FF increased significantly from 55% to 73%, efficiency increased from 13.5% to 16.5%, and V_oc_ was 12.39 V. Figure 6a,b show the schematic diagram and implementation diagram of the module device (P1, P2, P3) structure, respectively.

**Figure 7:**
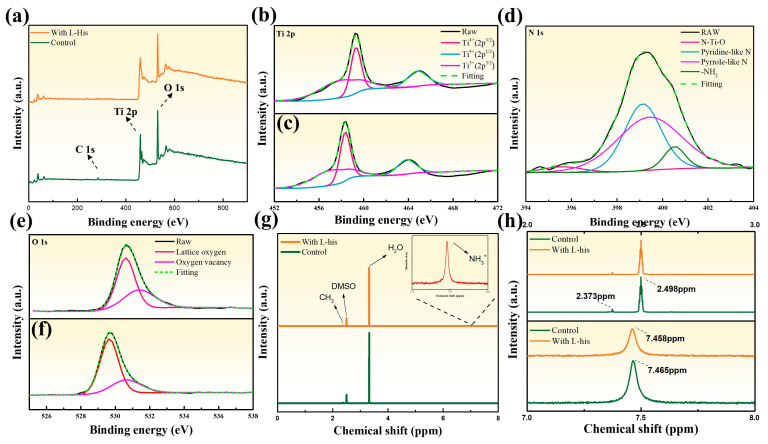
(**a**) XPS spectra of transport layer/perovskite interface with and without L-His. Fitting curves of Ti 2p (**b**) without L-His, (**c**) with L-His. Fitting curves of N 1s (**d**) with L-His. Fitting curves of O 1s (**e**) without L-His and (**f**) with L-His. (**g**,**h**) Nuclear magnetic resonance (^1^H NMR) analysis of transport layer/perovskite interface with and without L-His.

We utilized XPS to further investigate the possible reaction phenomena in TiO_2_/L-His films. The XPS spectra of TiO_2_ and TiO_2_/L-His for Ti 2p, N 1s, and O 1s are shown in Figure 7a. Before analysis, the spectra were calibrated using the C 1s peak (284.4 eV). Figure 7b,c display the high-resolution Ti 2p XPS spectra of TiO_2_ and TiO_2_/L-His, respectively. Changes in the peak integration area indicate changes in stoichiometry. After the introduction of L-His, the overall peak area of Ti^4+^ increased from 51.24% to 57.57%, while the overall peak area of Ti^3+^ decreased from 48.76% to 42.43%. The introduction of L-His leads to a coordination mechanism with Ti^4+^ ions, enhancing the stability of Ti^4+^ and preventing its reduction to lower valence states (Ti^3+^). The peak areas of the three peaks at 458.34 eV, 463.84 eV, and 457.1 eV are listed in Table S4. Besides observing changes in the peak area of Ti 2p, a slight shift in binding energy was also noted, indicating the impact of L-His on the electronic state of Ti elements [31]. The reduced binding energy is due to L-His carboxyl groups bonding with TiO_2_, which passivates oxygen vacancies since the oxygen vacancies on the TiO_2_ surface tend to bind free electrons, leading to non-radiative recombination and a decrease in PCE.

Figure 7d shows the fitted XPS spectrum of the N 1s orbital, with peaks at 395.61 eV, 399.13 eV, 399.42 eV, and 400.53 eV corresponding to N-Ti-O, pyridine-like N, pyrrole-like N, and -NH2 peaks, respectively. These fitted peaks indicate the deposition of L-His on TiO_2_ [32,33], with the fitting data listed in Table S5. Figure 7e,f further analyze the high-resolution O 1s XPS spectra of TiO_2_ and TiO_2_/L-His samples. Independent peaks were fitted at 529.65 eV and 530.62 eV, corresponding to lattice oxygen (O_I_) and vacancy oxygen (O_II_), respectively, with the fitting data listed in Table S6. The figures clearly show that after adding L-His, the peak area of vacancy oxygen decreased from 38.25% to 28.01%, and the ratio of vacancy oxygen to lattice oxygen derived from the fitted peaks of the O 1s spectrum decreased from 0.619 to 0.389, indicating a significant reduction in oxygen vacancies in the lattice.

The XPS spectra of L-His soaked for 24 h are shown in Figure S6. The Ti 2p spectrum shows a decrease in the Ti^4+^ peak area, with some Ti^4+^ reduced to Ti^3+^ ions. Ti^3+^ can create shallow traps below the conduction band and capture electrons migrating to the conduction band [34]. Excessive L-His induces H dissociation into protons, which bind lattice oxygens, generating Ti^3+^ species [35]. A schematic representation of the defect energy levels in the TiO_2_ transport layer is shown in Figure S7. The O 1s spectrum shows an increase in oxygen vacancies from 28.01% to 38.71%. XPS O1s spectra might include contributions from atmospheric atomic components. Oxygen vacancies in TiO_2_ can adsorb carbon- and oxygen-containing contaminants from the atmosphere, leading to an overall increase in peak intensity. Furthermore, the -COOH functional group in L-His contains oxygen, which may result in higher-than-theoretical oxygen vacancy concentrations. These adsorbates and the -COOH group modify the purity of the TiO_2_/perovskite interface, although they do not prevent the formation of strong bonds between the -COOH group and TiO_2_. Nonetheless, the trend still shows a significant reduction in oxygen vacancies in TiO_2_ upon the introduction of L-His [36,37]. The N 1s spectrum shows a decrease in pyridine-like N from 37.28% to 28.20% and pyrrole-like N from 52.21% to 34.78%. Therefore, when the deposition time exceeds 5 min, the increase in oxygen vacancies and Ti^3+^ leads to more defects in the film. This causes higher non-radiative recombination and reduced carrier transport ability, consequently reducing the short-circuit current.

**Figure 8:**
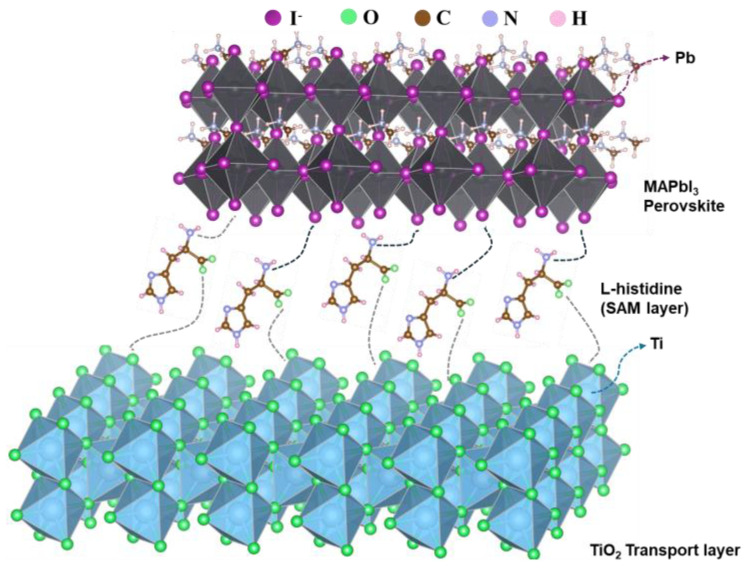
Chemical interactions of self-assembled monolayer at interface between perovskite and TiO_2_ transport layers.

Figure 7g presents proton nuclear magnetic resonance (^1^H NMR) analysis to investigate charge transport performance. Initially, ^1^H NMR measurements were used to verify the interactions between L-His and the perovskite. Besides studying the bonding interactions between L-His and the transport layer, the interfacial bonding and energy-level matching between the perovskite absorber layer and L-His are also crucial. Figure 8 shows a schematic diagram of the interactions of L-His at the interface with the electron transport layer and the perovskite layer. Introducing L-His not only enhances the wettability of the TiO_2_ surface but also passivates defect states in the perovskite film. It strengthens the interaction between the electron transport layer and the perovskite interface, significantly improving the device’s aging performance. Further analysis was conducted on the liquid spectra of the perovskite precursor solution with and without L-His. The signal at δ = 3.321 ppm corresponds to water, while the signal at approximately δ = 2.502 ppm corresponds to the solvent DMSO. Figure 7h further enlarges the 2 to 3 ppm region, revealing a signal at δ = 2.374 ppm attributed to the methylamine CH_3_ group and a resonance signal at δ = 7.4648 ppm corresponding to NH^3+^ [38].

When L-His is added, the characteristic peak (δ = 2.373 ppm) does not show a significant shift, indicating no changes in the chemical structure. However, after adding L-His to the perovskite solution, a slight shift is observed in the NH^3+^ peak (δ = 7.4648 ppm). The signal shifts to a lower field by approximately 0.007 ppm, attributed to the bonding between hydrogen in the L-His structure and the perovskite halides, leading to a de-shielding effect. This can be corroborated by electrical property analysis, which confirms the enhancement in charge transport performance [39].

## 4. Conclusions

In this study, L-His was introduced at the interface between the electron transport layer (TiO_2_) and perovskite. SEM analysis revealed that the surface morphology of the perovskite film became denser. From the PL and TRPL spectra, an improvement in the carrier extraction ability of the transport layer was observed. The average carrier lifetime decreased from 56.4 ns to 24.4 ns, indicating faster carrier extraction into the transport layer and reduced radiative recombination. The bonding between hydrogen in L-His and the perovskite is believed to enhance carrier transport properties. XPS results showed that L-His suppressed oxygen vacancies in the lattice, and the increased Ti^4+^ content promoted a passivation effect.

In solar cell devices, the addition of L-His significantly increased the FF, which contributed to the suppression of interface defects, improved film quality, and enhanced performance. The FF increased from 72% to 79%, and the PCE improved from 15.8% to 17.3%. After 500 h of aging tests, the PCE maintained 91% of its original efficiency. This result indicates that the hydrogen atoms in the imidazole ring of L-His provide lone-pair electrons, passivating uncoordinated Pb^2+^ defects in the perovskite film. Furthermore, the -NH_2_ groups of L-His can form hydrogen bonds with MA ions, enhancing the stability and aging characteristics of the perovskite film. Finally, integrating L-His into a large-area module (5 cm × 10 cm) increased the FF from 55% to 73%, and the PCE reached 16.5% for a 25 cm^2^ module. These results demonstrate that the introduction of L-His not only enhances device performance but also has the potential for the development of large-scale perovskite solar modules.

## Figures and Tables

**Table 1:** Photovoltaic performance of perovskite solar cells with and without L-His.

Structure/Device	J_sc_ (mA/cm^2^)	V_oc_ (V)	FF (%)	PCE (%)
Control (single cell)	21.45	1	75	16.26
With L-His (single cell)	21.51	1.01	79	17.30
Control (module)	1.97	12.30	55	13.56
With L-His (module)	1.82	12.39	73	16.54

## Data Availability

Data are contained within the article or supplementary material.

## Supplementary Materials

The following supporting information can be downloaded at: https://www.mdpi.com/article/10.3390/nano14151315/s1, Figure S1: SEM images of perovskite films deposited on L-His with different concentration: (a) control, (b) 0.02 mM, (c) 0.04 mM, (d) 0.06 mM, (e) 0.6 mM. Figure S2: The contact angel of perovskite precursor (a) without (control) and (b) with L-His. Figure S3: Photovoltaic performance of perovskite solar cells with different L-His deposition times. Figure S4: (a) I-V characteristics of the perovskite solar cell at different concentration of L-His. (b) I–V curve of perovskite solar cell. (c) Transmittance of TiO_2_ layer with and without L-His (control). Figure S5: Dark current of perovskite devices soaking L-his 24 h and without L-his (control). Figure S6: (a) XPS spectra of Ti 2p (b) N 1s O 1s transport layer/perovskite interface fitting curves with soaking L-his 24 h. Figure S7: Schematic representation of the defect energy levels in the TiO_2_ transport layer. Table S1: Summary of different SAM Materials. Table S2: Trap-Filling Limited Voltage (V_TFL_) and trap density (N_t_) calculation of different electron transport layer from SCLC measurement. Table S3: Fitting parameters of TRPL spectra for perovskite with and without L-His. Table S4: XPS fitting data of Ti 2p with and without L-His. Table S5: XPS fitting data of N 1s with and without L-His. Table S6: XPS fitting data of O 1s with and without L-His.
