# Supplementary material for: Large-Area Perovskite Solar Module Produced by Introducing Self-Assembled L-Histidine Monolayer at TiO2 and Perovskite Interface
Source: Nanomaterials (Basel). 2024 Aug 4;14(15):1315. doi: 10.3390/nano14151315 (PMC11314024; doi:10.3390/nano14151315)
Supplement: Supplementary file 1 [file nanomaterials-14-01315-s001.zip › nanomaterials-3112898-supplementary.pdf]

Supplementary information

Large-area Perovskite Solar Module Produced by Introducing Self-Assembled L-Histidine Monolayer between TiO_2 and Perovskite Interface

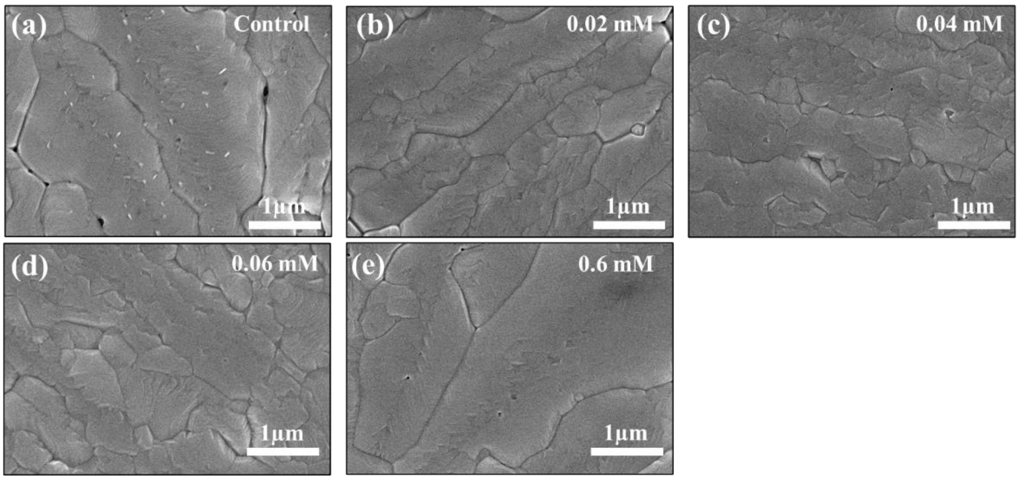

Figure S1. SEM images of perovskite films deposited on L-His with different concentration: (a) control, (b) 0.02mM, (c) 0.04mM, (d) 0.06mM, (e) 0.6mM.

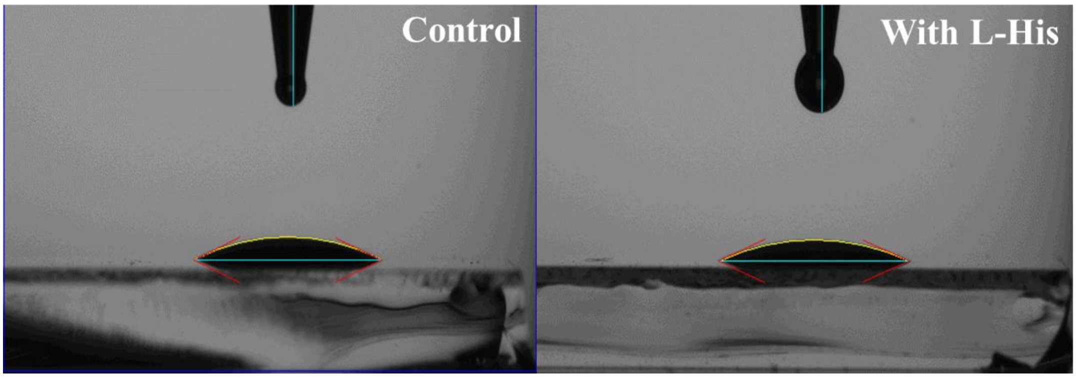

Figure S2. The contact angel of perovskite precursor (a) without (control) and (b) with L-His.

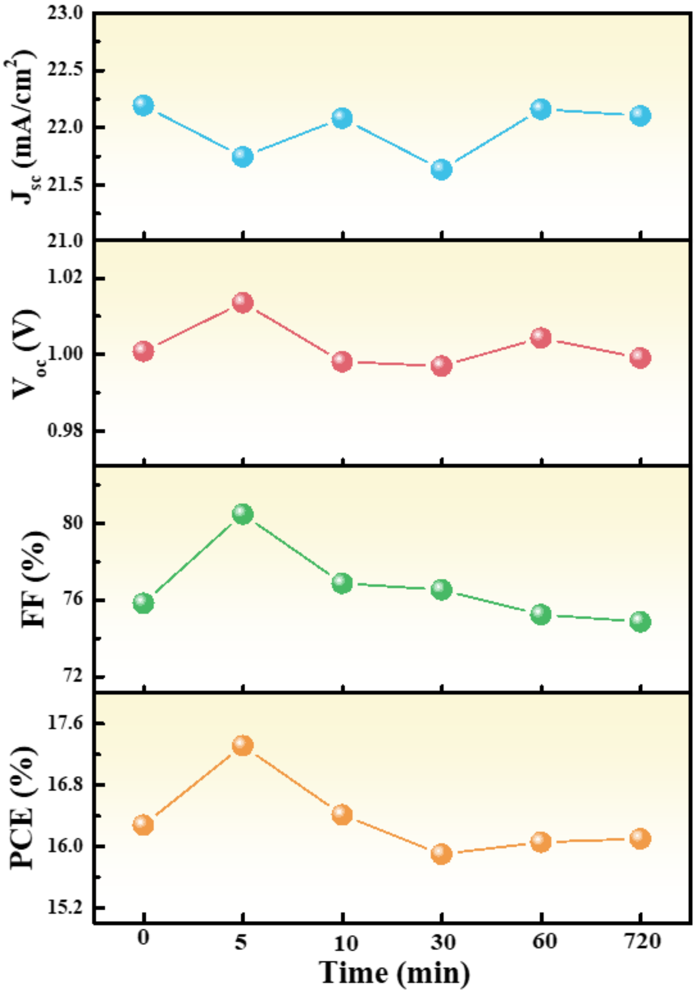

Figure S3. Photovoltaic performance of perovskite solar cells with different L-His deposition times.

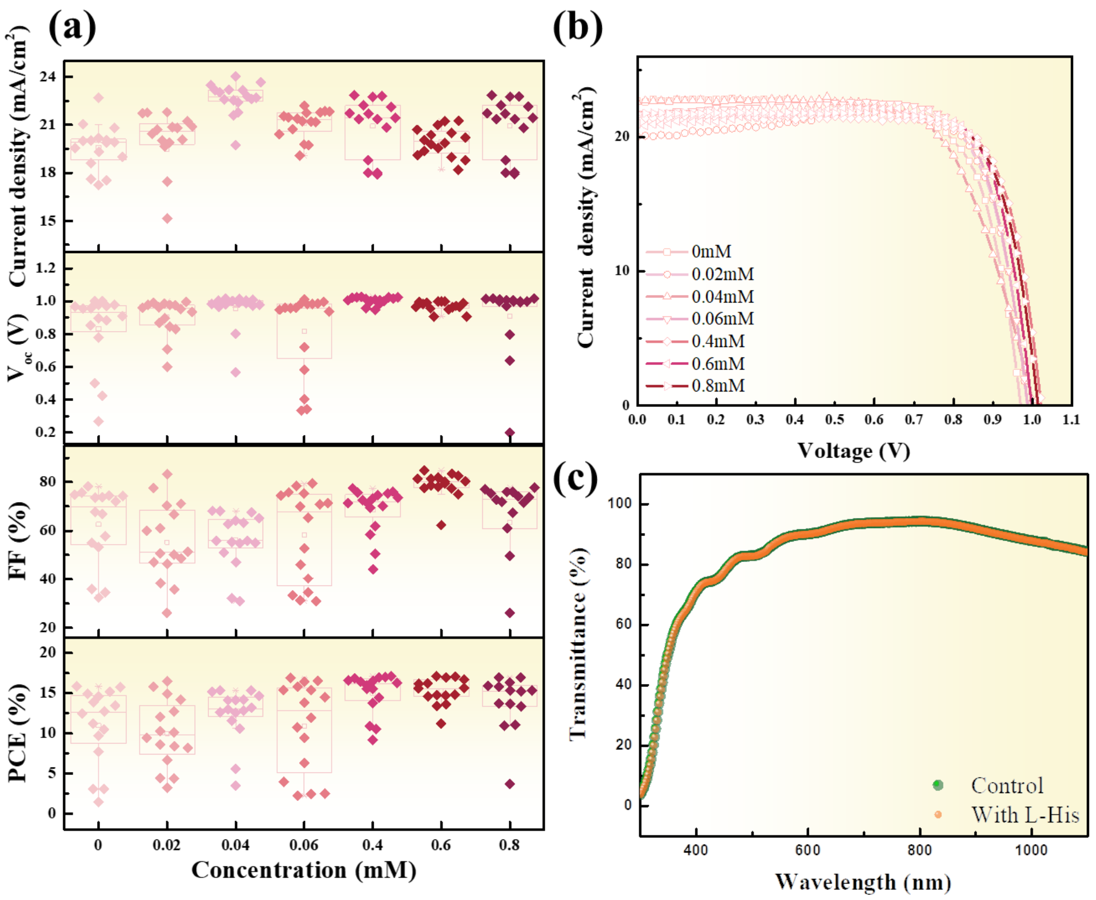

Figure S4. (a) I-V characteristics of the perovskite solar cell at different concentration of L-His. (b) I-V curve of perovskite solar cell. (c) Transmittance of TiO_2 layer with and without L-His (control).

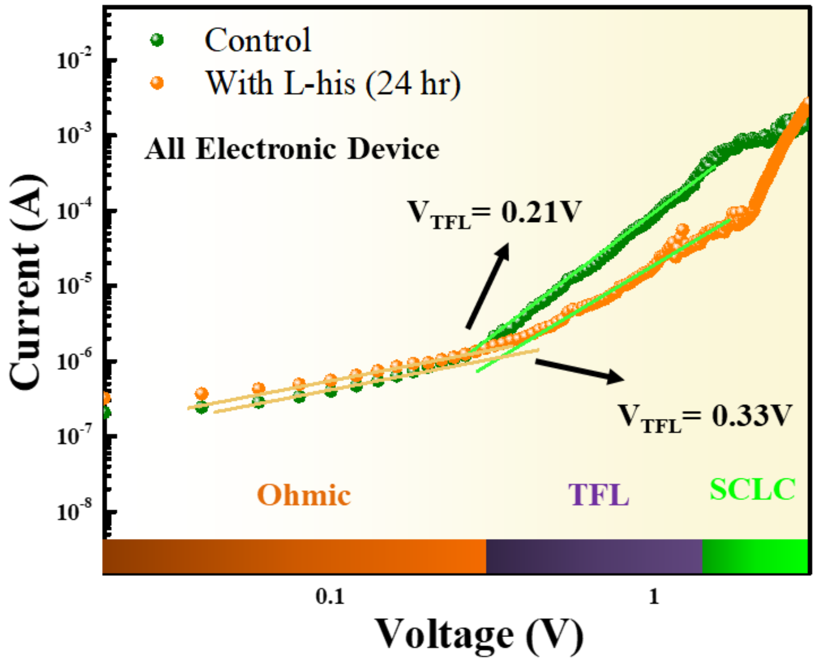

Figure S5. Dark current of perovskite devices soaking L-his 24 hours and without L-his (control).

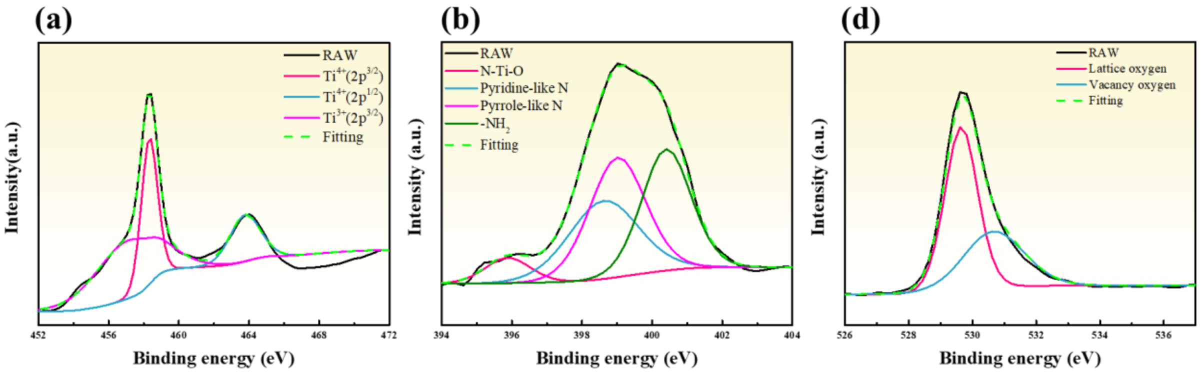

Figure S6. (a) XPS spectra of Ti 2p (b) N 1s O 1s transport layer/perovskite interface fitting curves with soaking L-his 24 hours.

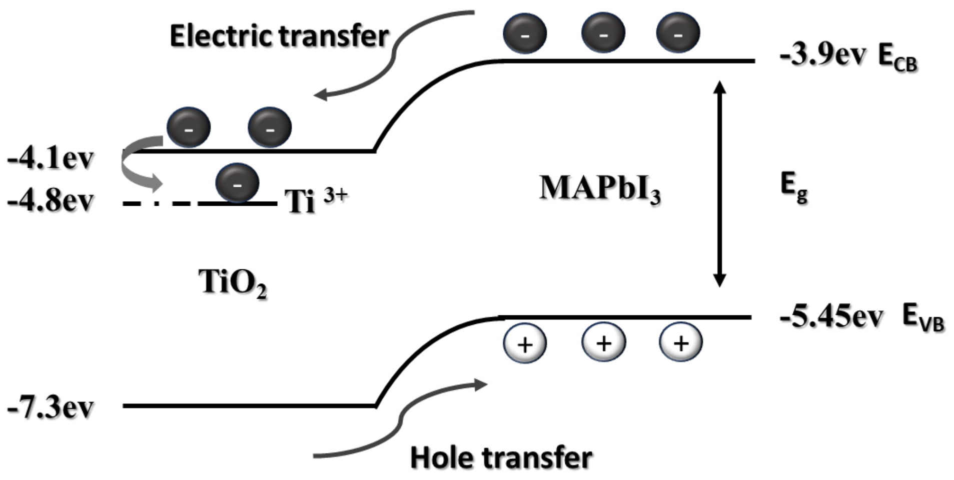

Figure S7. Schematic representation of the defect energy levels in the TiO_2 transport layer.

Table S1. Summary of different SAM Materials

SAM material	Solar cell	chemical properties	PSC performance	Reference
3-(Triethoxysilyl) propylamine (TSPA)	p-i-n (inverted)	The TSPA passivate hydroxyl group on the surface of the NiOx and improve defect passivation	20.21% (0.0464 cm^2)	[7]
Bifunctional 4-picolinic acid	n-i-p	The COOH group is chemically grappled with mp-TiO_2	18.9% (0.09 cm2)	[8]
glycine	n-i-p	Reducing the inevitable defects formed during crystallization of perovskite	12.02%	[9]
dopamine (DA)	n-i-p	Regulating growth and passivating surface trap states via N–H/I hydrogen-bonding interactions. The hydroxyl group reacts with SnO_2 to stabilize the perovskite structure.	16.87%	[23]
amino acid (AA)	p-i-n	The introduce of AAs into $MAPbI_3$-based PSCs resulting in reductions in oxygen-induced photodegradation	20.1%	[24]
glycine	n-i-p	The carboxylic acid group of glycine reacts with surface hydroxyl groups of SnO_2	20.68%	[18]
L-Histidine	n-i-p	The -NH_2 group can form hydrogen bonds with MA ions, while the carboxyl group can fill oxygen vacancies in the TiO_2.	16.54% (25 cm^2)	This work

Table S2. Trap-Filling Limited Voltage (V_{TFL}) and trap density (N_t) calculation of different electron transport layer from SCLC measurement.

**Structure**	**V_{TFL} (V)**	**N_t (cm^{-3})**
**Control**	**0.21**	**4.66×10^{15}**
**With L-His**	**0.17**	**3.96×10^{15}**

Table S3. Fitting parameters of TRPL spectra for perovskite with and without L-His

**Structure**	τ_{avg} ***(ns)***	τ_1 ***(ns)***	τ_2 ***(ns)***
**Control**	**56.42**	**68.58**	**12.26**
**With L-His**	**24. 40**	**35.08**	**9.19**

Table S4. XPS fitting data of Ti 2p with and without L-His

Structure	$Ti^{4+}(2p^{3/2})$	$Ti^{4+}(2p^{1/2})$	$Ti^{3+}(2p^{3/2})$
**Control**	**31.52%** **(459.30eV)**	**19.72%** **(464.79eV)**	**48.76%** **(457.95eV)**
**With L-His**	**37.87%** **(458.34eV)**	**19.70%** **(463.84eV)**	**42.43%** **(457.1eV)**
**With L-His (24 hours)**	**31.65%** **(458.31eV)**	**18.64%** **(463.77eV)**	**49.71%** **(457.12eV)**

Table S5. XPS fitting data of N 1s with and without L-His

Structure	N-Ti-O	Pyridine like-N	Pyrrole like-N	$-NH_2$
**With L-His**	**3.3%<br>(395.61V)**	**37.28%<br>(399.13eV)**	**52.21%<br>(399.42eV)**	**7.17%<br>(400.53eV)**
**With L-His<br>(24 hours)**	**5.96%<br>(395.88V)**	**28.20%<br>(398.62eV)**	**34.78%<br>(399.01eV)**	**31.07%<br>(400.40eV)**

Table S6. XPS fitting data of O 1s with and without L-His

Structure	Lattice oxygen	Oxygen vacancy
**Control**	**61.75%**<br>**(530.60eV)**	**38.25%**<br>**(531.34eV)**
**With L-His**	**71.99%**<br>**(529.65eV)**	**28.01%**<br>**(530.62eV)**
**With L-His**<br>**(24 hours)**	**61.29%**<br>**(529.64eV)**	**38.71%**<br>**(530.65eV)**
